# Cancer Organoids as reliable disease models to drive clinical development of novel therapies

**DOI:** 10.1186/s13046-024-03258-7

**Published:** 2024-12-28

**Authors:** Giovanni Blandino, Ronit Satchi-Fainaro, Ingeborg Tinhofer, Giovanni Tonon, Sarah C. Heilshorn, Yong-Jun Kwon, Ana Pestana, Carlotta Frascolla, Luca Pompili, Aurora Puce, Sara Iachettini, Annalisa Tocci, Sofia Karkampouna, Marianna Kruithof-de Julio, Piera Tocci, Nicla Porciello, Klizia Maccaroni, Daniela Rutigliano, Xiling Shen, Gennaro Ciliberto

**Affiliations:** 1 Translational Research Unit, IRCCS Regina Elena National Cancer Institute, Rome, Italy; 2 Deapartment of Physiology and Pharmacology, Tel Aviv University, Tel Aviv, Israel; 3 Department of Radiooncology and Radiotherapy, Charité University Medicin, Berlin, Germany; 4 Center of Omics Sciences, IRCCS San Raffaele Scientific Institute, Milan, Italy; 5 Department of Materials Science and Engineering, Stanford University, Stanford, USA; 6 Luxembourg Institute of Health, Strassen, Luxembourg; 7 Cellular Network and Molecular Therapeutic Target Unit, IRCCS Regina Elena National Cancer Institute, Rome, Italy; 8 Tumor of Immunology and Immunotherapy Unit, IRCCS Regina Elena National Cancer Institute, Rome, Italy; 9 Department for BioMedical Research, University of Bern, Swiss, Switzerland; 10 Unit of Preclinical Models and New Therapeutic Agents, IRCCS Regina Elena National Cancer Institute, Rome, Italy; 11 GI Medical Oncology, MD Anderson Cancer Center, Houston, TX USA; 12 Terasaki Institute of Biomedical Innovation, Los Angeles, CA USA; 13 Scientific Direction, IRCCS Regina Elena National Cancer Institute, Rome, Italy

**Keywords:** Organoid, Precision medicine, Patient-derived 3D culture model, Targeted therapy, Preclinical models, Cancer spheroid

## Abstract

On September 23–24 (2024) the 6th Workshop IRE on Translational Oncology, titled “Cancer Organoids as Reliable Disease Models to Drive Clinical Development of Novel Therapies,” took place at the IRCCS Regina Elena Cancer Institute in Rome. This prominent international conference focused on tumor organoids, bringing together leading experts from around the world.

A central challenge in precision oncology is modeling the dynamic tumor ecosystem, which encompasses numerous elements that evolve spatially and temporally. Patient-derived 3D culture models, including organoids, explants, and engineered or bioprinted systems, have recently emerged as sophisticated tools capable of capturing the complexity and diversity of cancer cells interacting within their microenvironments. These models address critical unmet needs in precision medicine, particularly in aiding clinical decision-making. The rapid development of these human tissue avatars has enabled advanced modeling of cellular alterations in disease states and the screening of compounds to uncover novel therapeutic pathways.

Throughout the event, distinguished speakers shared their expertise and research findings, illustrating how organoids are transforming our understanding of treatment resistance, metastatic dynamics, and the interaction between tumors and the surrounding microenvironment.

This conference served as a pivotal opportunity to strengthen international collaborations and spark innovative translational approaches. Its goal was to accelerate the shift from preclinical research to clinical application, paving the way for increasingly personalized and effective cancer therapies.

## Drug screening in tumor organoids

*Giovanni Blandino* (IRCCS, Regina Elena National Cancer Institute, Rome, IT)

In the field of oncologic clinical research, tumor organoids have emerged as some of the most promising models [1]. Patient-derived endometrial cancer organoids (EC_PDOs) are patient-specific, three-dimensional (3D) cellular models that effectively capture tumor heterogeneity, as well as crucial cell-to-cell and cell-to-microenvironment interactions. Notably, EC_PDO cultures also retain stromal cells—a vital component of the tumor microenvironment that plays a key role in tumor progression, metastasis, recurrence, and response to chemotherapy [2].

**Fig. 1:**
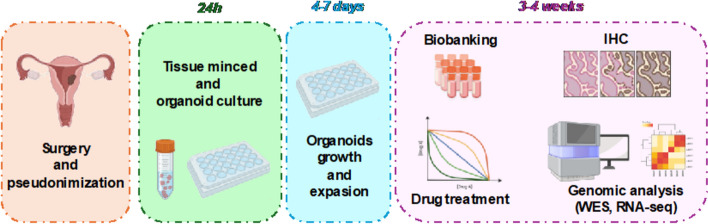
Endometrial Cancer Organoids Workflow

By faithfully replicating the histological and molecular features of the corresponding tumor and preserving various populations of tumor tissue, these models hold significant promise. They enable in vitro replication of the original tissue’s characteristics and provide a powerful platform for predicting pharmacological responses in a more translational, clinically relevant manner (Fig. 1).

## 3D-Bioprinted cancer models for target discovery, drug development, and personalized therapy

*Ronit Satchi-Fainaro* (Tel Aviv University, IL). Many drugs show promising results in laboratory research, but eventually fail in clinical trials. We hypothesize that one main reason for this translational gap is that the cancer models used are inadequate. Most models lack the complex tumor-stromal cell interactions with their microenvironment which are required for tumor progression. Conventional 2D cultures, where cells grow on rigid plastic plates mainly as mono-cultures of a single type of cells, are not able to recapitulate the complex settings of such interactions. Therefore, there is a need to develop a 3D model that better mimics the tumor microenvironment [3]. Hence, we developed a vascularized, hydrogel-based 3D-bioprinted tumor model consisting of patient-derived tumor, microenvironmental, and immune cells [4]. Our 3D-bioprinted models are based on a library of hydrogels that we developed as scaffolds for different tumor types, designed according to the mechanical properties of the tissue/organ of origin. The patient-derived models consist of cells from a biopsy, and include functional and perfusable vessels that allow serum and peripheral blood mononuclear cells (PBMC) to flow when connected to a pump. Using this unique model platform, we identified P-selectin as a novel immune checkpoint in the brain regulating cancer-microglia-tumor-associated macrophages (TAMs) interactions [5]. Based on this finding, we have begun an investigator-initiated clinical trial, testing the efficacy of the anti-P-selectin antibody, Crizanlizumab, alone or in combination with anti-PD-1 antibody, Nivolumab, for glioblastoma and melanoma brain metastasis patients (NCT05909618). Moreover, we are currently validating our 3D platform for its ability to mimic patient-specific tumors and their microenvironment in order to predict patient response to different treatments (such as chemotherapy, immunotherapy, and targeted therapies). In addition, we exploit this platform to identify unique biomarkers, which can be used as targets for our Turn-ON chemiluminescent probes for image-guided surgery, early detection, and/or companion diagnostics [6]. Hence, we are currently conducting an 80-patient “basket” clinical trial testing different treatments on patient samples to validate the ability of our model to predict patient outcomes covering 7 different cancer types. These unique 3D-bioprinted models have the potential to facilitate target discovery and drug development, as well as to serve as a reliable system for precision medicine.

## Advances of Patient-Derived Organoids on Personalized Radiotherapy

*Ingeborg Tinhofer-Keilholz* (Charite University Hospital Berlin, Department of Radiooncology and Radiotherapy)

**Fig. 2:**
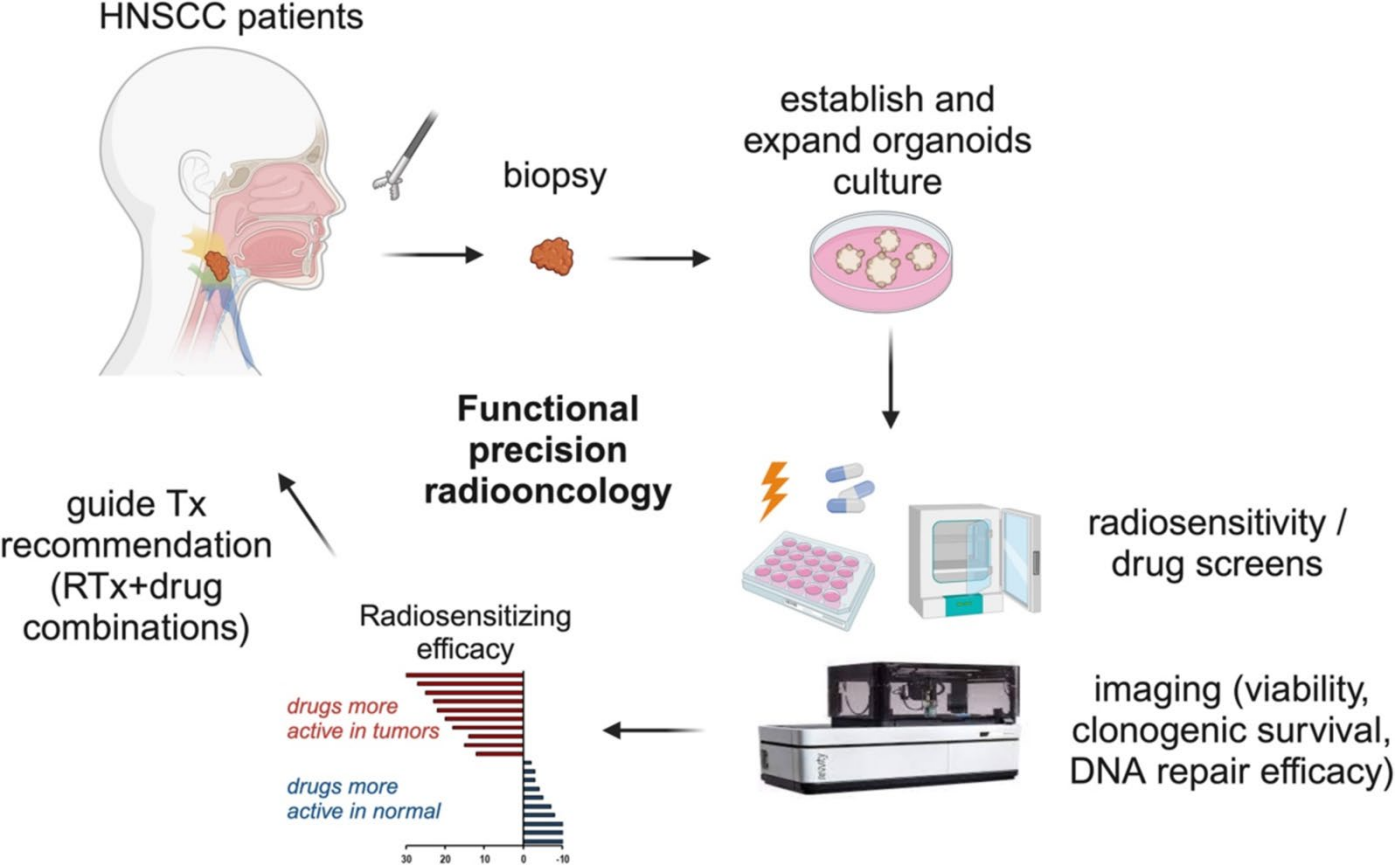
Functional Precision Radio-oncology in HNSCC. The schematic illustrates the potential workflow of functional precision radio-oncology. Tissue samples (tumour and adjacent normal tissue) are collected during diagnostic biopsy or curative-intent surgery. Following the establishment and expansion of organoid cultures, screening is conducted for single and combination treatment regimens. Experimental treatment efficacy is assessed using read-outs such as cell viability, clonogenic survival, and/or residual DNA double-strand breaks. Drugs are ranked based on their efficacy scores in tumour and normal organoids. Top candidate therapies are subsequently reported to molecular tumour boards to inform treatment recommendations. *Figure created with biorender.com*

Utilising patient-derived organoids (PDOs) for radiosensitivity and drug screening offers significant potential in guiding treatment decisions [7]. This approach to functional precision oncology (Fig. 2) may be particularly relevant in head and neck squamous cell carcinoma (HNSCC) – a disease characterised by the absence of druggable driver mutations [8], pronounced interpatient heterogeneity [9], and a lack of predictive biomarkers [10]. To advance our understanding of resistance mechanisms in tumour stem cells, we have made substantial investments in developing advanced preclinical models derived from stem cells within bulk tumour tissue. These efforts have enabled us to establish one of the world’s largest collections of PDO models [11]. We demonstrated that PDO generation and expansion can be scaled for medium-throughput ex vivo drug screening in most HNSCC patients, provided stringent measures are implemented to ensure high sample quality and prompt tissue processing [11]. Furthermore, our findings indicate that PDO engraftment correlates with poor overall survival, underscoring the potential of these models to unravel resistance mechanisms and identify novel combination therapies for patients at high risk of treatment failure following standard treatment.

## A 3D culture platform for high-throughput drugs screening, multidimensional omics and imaging assays on patient-derived organoids

*Giovanni Tonon* (IRCCS San Raffaele Scientific Institute, Milan, Italy)

**Fig. 3:**
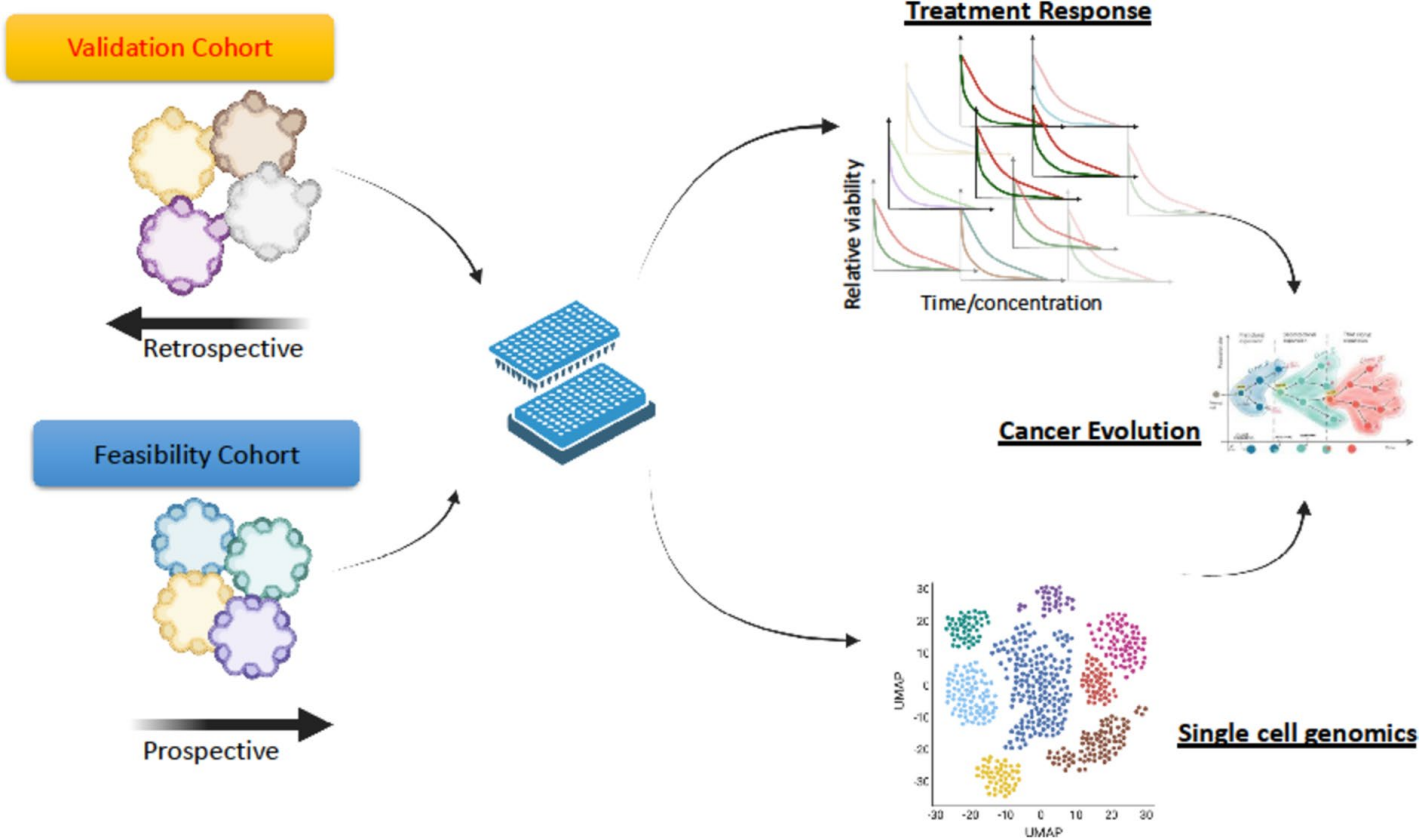
Workflow from high-throughput screenings in a microfluidic PDO platfoform towards single cell multi-omic analyses

Patient-derived organoids (PDOs) are becoming central in the clinical practice, to preemptively identify the optimal treatment for each patient. However, the implementation of PDOs have been hampered by several bottlenecks including sample requirements and assay time. We have implemented a microfluidic-based device that miniaturises and greatly simplifies PDO cultures in a 384-plate format. Both retrospective and prospective clinical studies demonstrated its predictive value and its implementation in the clinical setting. Furthermor, our microfluidic platform allows subcellular phenotypic screenings, target engagement for the assessment of the efficacy of targeted therapies, alongside the ability to comprehensively define the genomic, epigenetic, transcriptomic, proteomic, lipidomic and metabolomic landscape of each PDO [12, 13] (Fig. 3).

## Advancing tumoroid models through bioprinting and biomaterials design

*Sarah C. Heilshorn* (Stanford University, Stanford, USA)

**Fig. 4:**
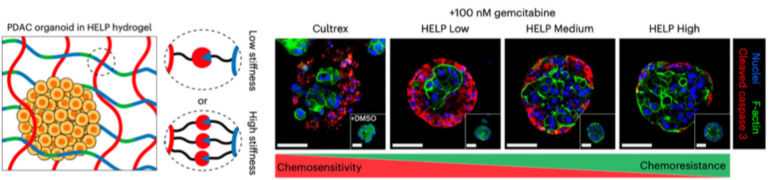
Left panel: Schematic of patient-derived pancreatic ductal adenocarcinoma (PDAC) organoid grown within an engineered matrix (termed a HELP hydrogel). Changing the number of chemical crosslinks between the biopolymers within the HELP hydrogel creates matrices with low, medium, and high stiffness. Right panel: PDAC organoids proliferated and grew similarly well within a commercially available Cultrex matrix or the engineered HELP hydrogels of Low, Medium, and High stiffness. Organoids grown in stiffer matrices (HELP High) became less sensitive to the chemotherapy gemcitabine, as shown by less immunostaining for the apoptosis marker cleaved caspase 3. Reprinted with permission (The figure is a modified version of a Fig. 1 in reference 16)

The phenotypic behavior of cancer cells is a consequence of both intrinsic factors, such as genetic mutations, and extrinsic factors, such as interactions with the tumor microenvironment (TME). One key component of the TME for solid tumors is the extracellular matrix (ECM), which becomes increasingly fibrotic and stiff upon disease progression [14]. Although ECM stiffness is often correlated with adverse clinical outcomes, most patient-derived cancer organoid cultures use commercially available matrices that are mechanically weak and do not mimic the stiffness of the parent tumor [15]. To address this limitation, Heilshorn et al*.* designed an engineered matrix composed of biopolymers upregulated in pancreatic ductal adenocarcinoma (PDAC) with stiffness spanning that of healthy and cancerous pancreatic tissue (Fig. 4) [16]. They discovered that PDAC organoids grown in stiffer matrices acquired chemoresistance to gemcitabine over time in vitro due to an upregulation of efflux transporters. Furthermore, they demonstrated that matrix mechanosignaling through CD44 cell-surface receptors was required for this effect, and that disruption of matrix mechanosignaling could resensitize PDAC organoids to chemotherapy. Together, these results demonstrate that designer matrices can be used to mimic aspects of the tumor TME for mechanistic studies of cancer.

## Accelerating Precision Medicine for Cancer Patients through Personalized Functional Profiling

*Yong-Jun Kwon* (Luxembourg Institute of Health, Luxembourg)

**Fig. 5:**
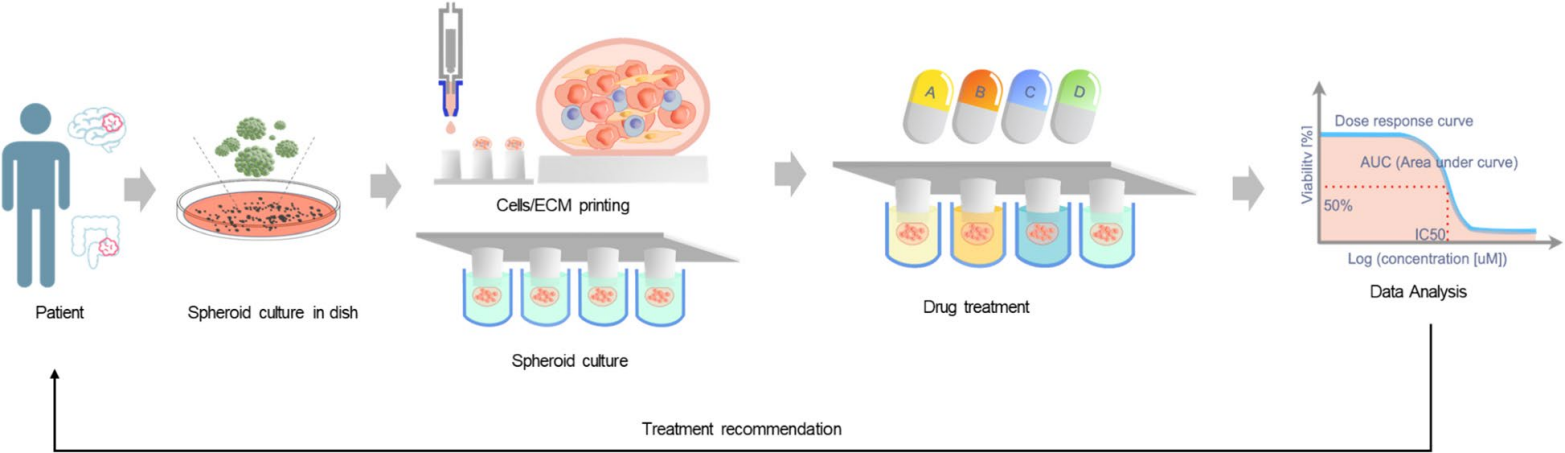
*The Personalized Functional Profiling (PFP) technology.* Surgical patient biopsies are dissociated and culturing tumor spheroid. Dissociated cells are printed onto a 3D-pillar support system using robot technology and grown as tumor spheroids. These tumor spheroids have the same genomic composition as the tumor of origin. Tumor spheroids are then challenged by a large panel of drugs (including FDA approved drugs). Growth (size of spheroids) is measured by an automated confocal microscope. To foster a comparative analysis of drug responses, we will employ an analysis of drug sensitivity, including the half-maximal inhibitory concentration (IC_50_) and the area under curve (AUC) of the dose–response curve (DRC). Drugs that show efficacy can potentially be reported to patients

A core principle of precision oncology is that genomic and molecular profiling of tumors enables the identification of the most effective therapies tailored to each patient [17, 18]. However, despite significant advancements in genomic technologies, predicting therapeutic success using computational approaches alone remains a challenge. To address this challenge, an integrated strategy that combines genomic analysis with direct drug response assessments using patient-derived cells (PDCs) offers a promising solution [19, 20]. This method allows for the evaluation of drug efficacy directly in vitro, providing treatment recommendations that better capture the intricate interactions between the tumor and therapeutic agents. Such a combined approach has the potential to refine treatment options by providing a more tailored strategy that takes into account the unique characteristics of each patient's tumor. The Luxembourg Institute of Health (LIH) has already explored this approach, known as Personalized Functional Profiling (PFP), through pilot clinical studies involving patients with metastatic colorectal cancer (mCRC) and recurrent glioblastomas (rGBM). These pilot studies focused on assessing the feasibility of PFP technology, demonstrating that it is possible to use this technology for personalized drug testing and treatment recommendations. Building on these positive outcomes, the current study aims to transition from feasibility testing to clinical validation, seeking to establish the broader effectiveness of PFP technology (Fig. 5).

## Preclinical models for rare tumors to advance precision oncology

**Fig. 6:**
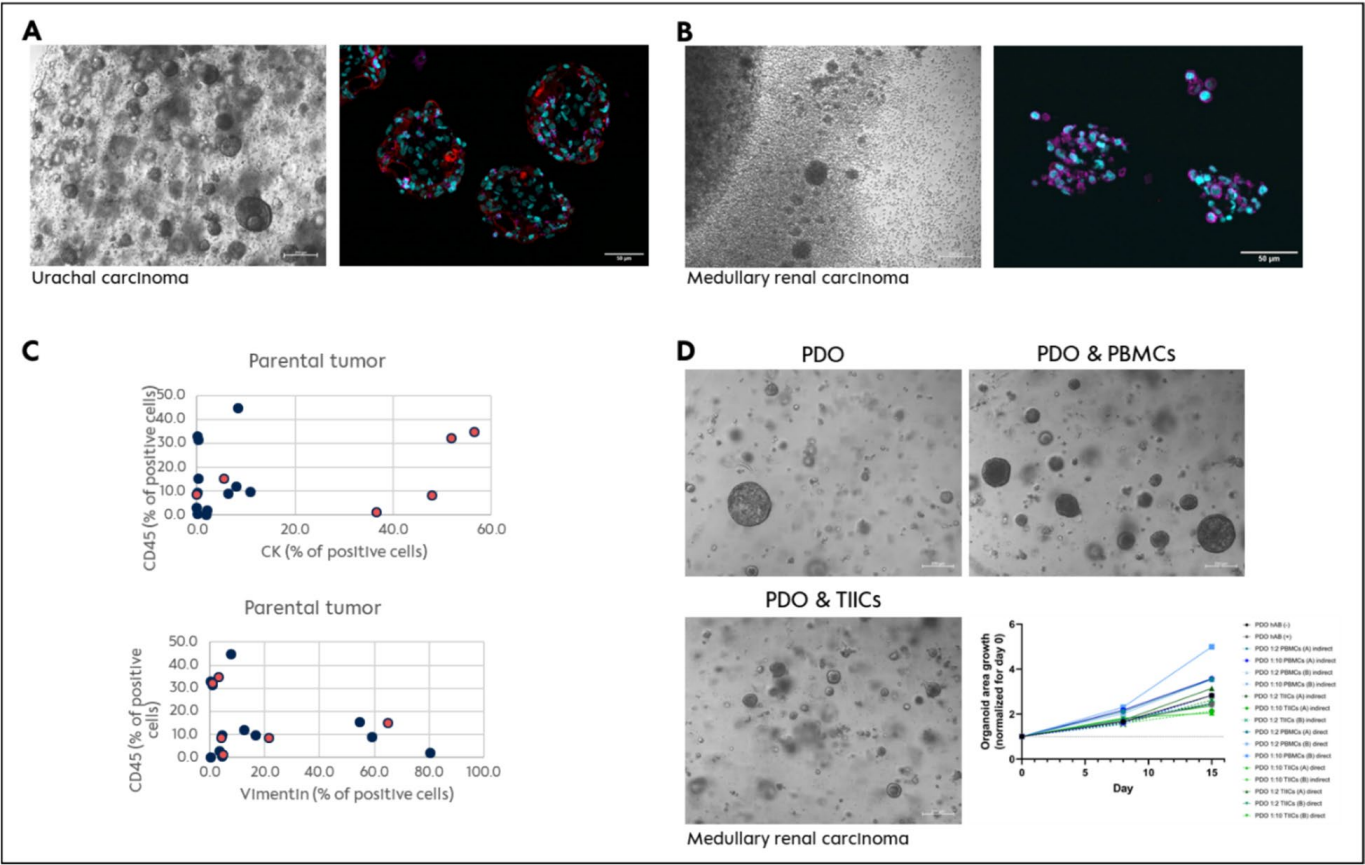
TIICs expansion and characterization. **A** Images representative of one Urachal carcinoma sample mPDO in brightfield and multiplex immunofluorescence (3D passage 1); depicted are cytokeration-7 (red), αSMA (green) and CD45 (magenta). **B** Images representative of one renal medullary carcinoma sample TIICs in brightfield and multiplex immunofluorescence (passage 1 and 3, respectively); depicted are cytokeration-7 (red), αSMA (green) CD45 (magenta). **C** Graphical representation of the proportion of cells positive for CD45, Vimentin and Cytokeratin within the parental tumor sample processed analysed via multiplex immunofluorescence. Coral colouring marks the tumours showing TIICs expansion. **D** Co-culture conditions tested for the medullary renal carcinoma cPDO with autologous PBMCs and TIICs with graphical representation of the organoid growth

*Ana Pestana* (Charité – Universitätsmedizin Berlin, Germany). Although rare by themselves, rare tumors (RT) constitute a significant public health problem and represent over 22% of all newly diagnosed cancers in Europe [21]. Due to the low incidence of the individual tumor types, clinical trials for specific molecular subtypes are difficult to perform, and there is a significant lack of preclinical models available to study novel treatment approaches, including precision oncology strategies and immunotherapy. Yet, experiences based on precision oncology programs show presence of targetable alterations in a relevant subset of patients, which needs preclinical evaluation for subsequent clinical development. Patient-derived organoids (PDO) are a powerful tool to mimic the tumor cells’ heterogeneity [22] while lacking the tumor microenvironment interactions [23], making them inadequate for preclinical evaluation of immunotherapy. Therefore, we dedicated our research/efforts to generate more advanced models modifying the classical PDO (cPDO) protocol to maintain the immune cell population in culture (mPDO). Eighteen RT samples (salivary gland cancer, medullary renal carcinoma, ameloblastoma, urachal carcinoma) were processed applying the mPDO protocol with 3/18 showing no growth, while we managed to yield 9/18 PDOs including native tumor infiltrating immune cells with a short life span (Fig. 6A) and 6/18 PDOs that were quickly overgrown by the native tumor infiltrating immune cells (Cs) (Fig. 6B). To understand the phenomenon of TIICs overgrowth in a few selected cases comprising benign and malignant tumors, the parental tissue was analyzed for tumor cell content using markers for fibroblasts (vimentin), epithelial (cytokeratin) and immune cells (CD45). Interestingly, TIICs overgrowth was independent of the proportion of immune cells in the native tissue, and was more frequently observed in tumors with low vimentin positive cells and high cytokeratin positive cells (Fig. 6C). To further gain insights into the biology of this population, cPDOs from the medullary renal carcinoma were generated and co-cultured with either autologous peripher blood mononuclear cells (PBMCs) or TIICs (Fig. 6D). Co-cultivation with PBMCs revealed a positive impact on PDO growth, while TIICs did not. When compared with autologous PBMCs, TIICs showed an increase in CD68, CD56 and CD8 positive cells. Current experiments aim to understand the mechanisms of TIICs activation, the characteristics causative for this expansion, as well as the possible role of TIICs as tool to predict immunotherapy response or as a therapy itself.

## Bladder-derived tumor organoid platform to test cancer precision medicine

*Carlotta Frascolla* (IRCCS, Regina Elena National Cancer Institute, Rome, IT)

**Fig. 7:**
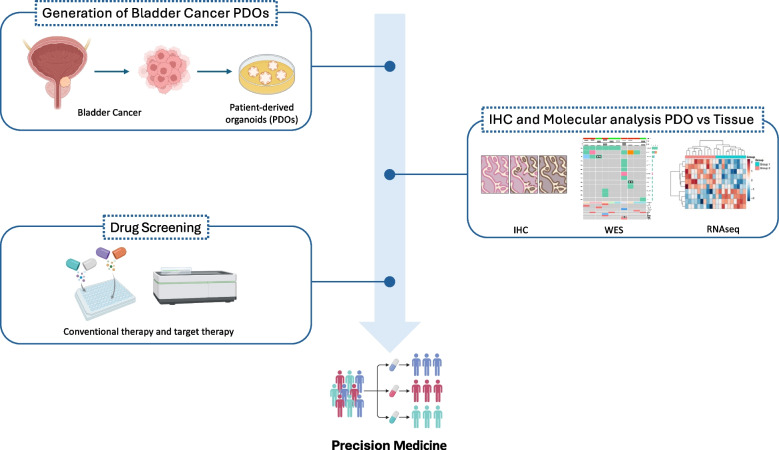
Bladder Cancer PDOs Workflow

High recurrence rate and variable treatment responses characterized bladder cancer (BLC) as one of the most common cancers globally. Despite advances in understanding its molecular mechanisms, developing effective therapies remains an un-met need for BLC patients. Standard treatments, as transurethral resection (TURBT), radical cystectomy (RC) and chemotherapy, often lead to unpredictable outcomes, even among patients with similar clinical profiles [24, 25]. This variability highlights the need to account for tumor complexity in personalizing therapies to improve survival. For addressing this issue, pre-clinical models able to accurately recapitulate inter- and intra-patients’ differences are crucial. We reported that bladder cancer patient-derived organoids (PDOs) provide a valuable tool for studying bladder cancer heterogeneity, offering promising preclinical models to explore drug responses and potentially improve personalized therapies (Fig. 7) [26, 27].

## Genetically engineered liver organoids as pre-clinical models in intrahepatic cholangiocarcinoma

*Luca Pompili* (IRCCS, Regina Elena National Cancer Institute, Rome, IT).

**Fig. 8:**
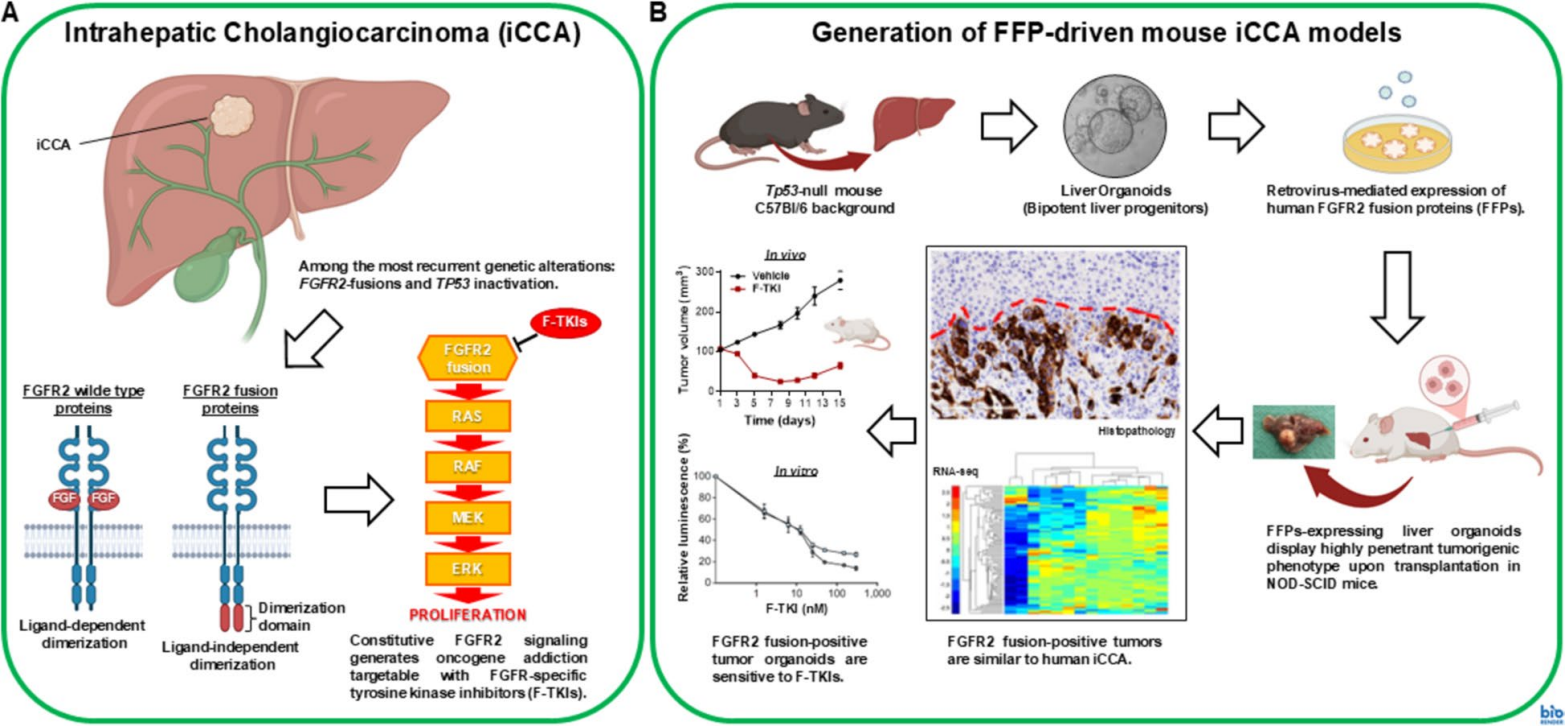
Schematic representation of FGFR2 fusion-positive intrahepatic cholangiocarcinoma (iCCA) (**A**) and generation of iCCA models based on murine liver organoids genetically modified to express FGFR2 fusion proteins (**B**)

Intrahepatic cholangiocarcinoma (iCCA) is a rare and hard to treat tumor with a grim prognosis. A subgroup of iCCA patients (about 15%) is characterized by FGFR2 fusion proteins (FFPs) expression [28] that usually co-occurs with inactivation of tumor suppressor genes such as *TP53*. Constitutive signaling triggered by FFPs drives and maintains tumorigenic transformation, thereby generating oncogene addiction vulnerable to FGFR-specific tyrosine kinase inhibitors (F-TKIs). Since the infrequence of iCCA, there is a paucity of genetically defined cellular and animal models suited to pre-clinical research on oncogene-directed therapies. Thus, to overcome this hurdle, we generated a mouse iCCA model based on ectopic expression of human FFPs in *Tp53*-null mouse liver organoids (C57Bl/6 background), which undergo oncogenic transformation upon intrahepatic transplantation in NOD-SCID immunodeficient mice [29]. These models recapitulate the histological, immunophenotypic and transcriptomic features typical of the human disease. Moreover, the respective tumor-derived organoids are sensitive to F-TKIs either in vitro or in vivo, thus conforming to the oncogene addiction paradigm. In aggregate, these results support our murine iCCA tumor organoids as models exploitable for further pre-clinical studies aimed to optimize the therapeutic strategies for FFPs-positive iCCA patients (Fig. 8).

## Developing 3D-printed Osteosarcoma models to comprehensively characterize molecular traits and evaluate the efficacy of novel therapeutic strategies

*Aurora Puce* (IRCCS, Regina Elena National Cancer Institute, Rome, IT).

**Fig. 9:**
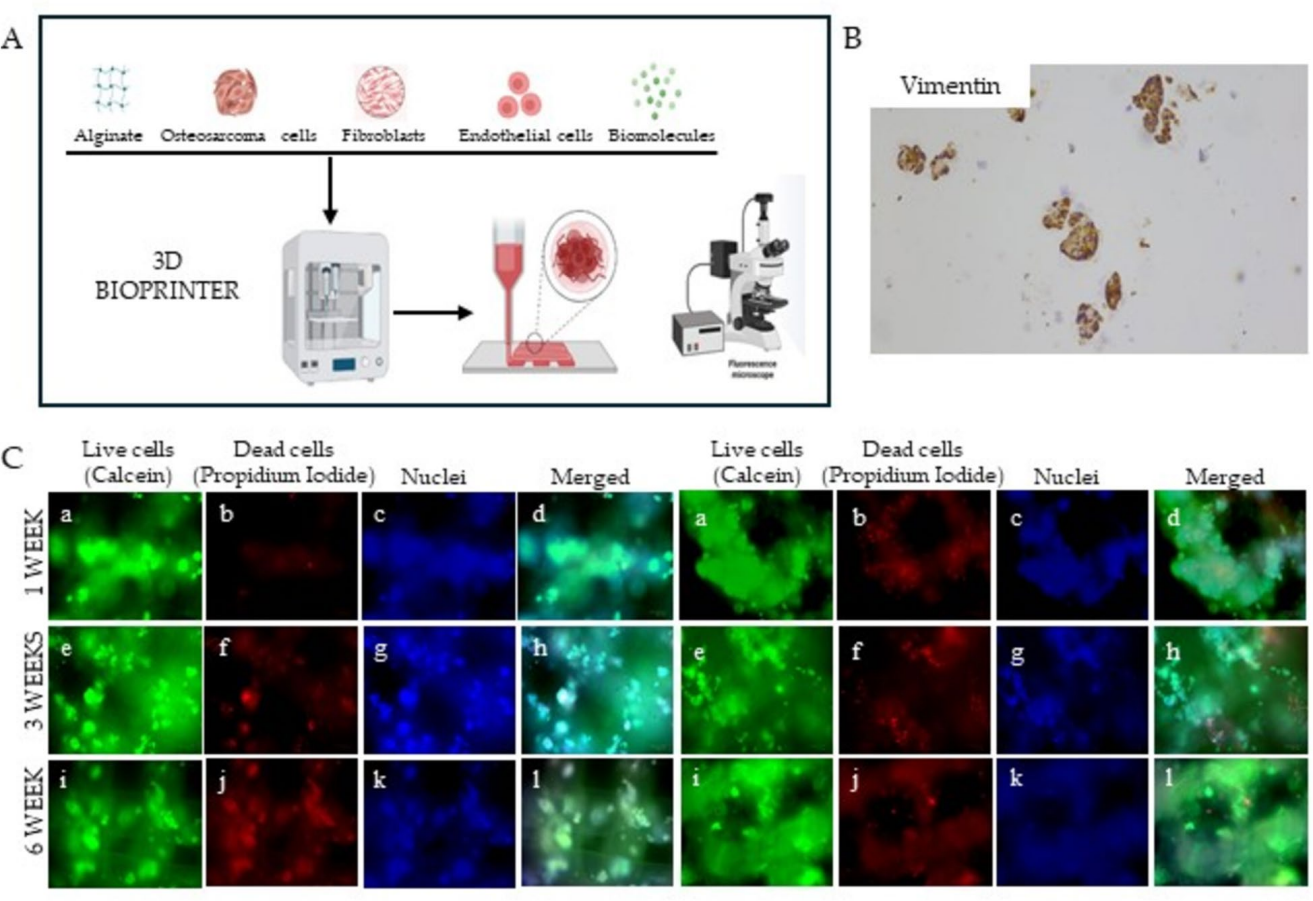
**A** Diagram of 3D extrusion based bioprinter working process: biomaterial, populations of interest and biomolecules are inserted into the instrument and extruded together in a software-aided shape. **B** Immunohistochemistry analysis conducted on 3D OS bioprinted models showing the positivity for Vimentin (magnification 100X). **C** 3D OS models were bioprinted starting from Sa-Os cell line alone (on the left) or in combination with fibroblasts (on the right). The models were cultured in a 6-well chamber slide and stained with the live-dead assay kit (Merck). The rows show live cell OS models stained with (a, e, i) Calcein-AM, (b, f, j) propidium iodide, (c,g,k) Hoechst 33,342 and (d,h,l) merged image

Osteosarcoma (OS) is the most common malignant bone tumor, with a worlwide incidence of 1–3 cases per million people annually [30]. Currenly treatment relies only on antiblastic drugs. The lack of targeted therapies is partly due also to the absence of standardized three-dimensional (3D) models which accurately replicate OS pathogenesis and allow to perform reliable in vitro studies. The tumor niche of OS in vivo is characterized by vasculature, stromal cells, and a 3D hypoxic mass with invasive margins [31]. In this study, we employed a 3D extrusion-based bioprinter to realise osteosarcoma models, using alginate as the bioink and three commercial OS cell lines (MG-63, Sa-OS, 143-B), either alone or in combination with fibroblasts. The models demonstrated full survival for up to six weeks, as confirmed by viability assay. Furthermore, immunohistochemistry analysis revealed that these OS models displayed a high degree of morphological similarity to patient tumors (Fig. 9).

## Cancer spheroids as reliable models for drug screening: functional characterization of a new class of G-quadruplex ligands

*Sara Iachettini* (IRCCS, Regina Elena National Cancer Institute, Rome, IT).

**Fig. 10:**
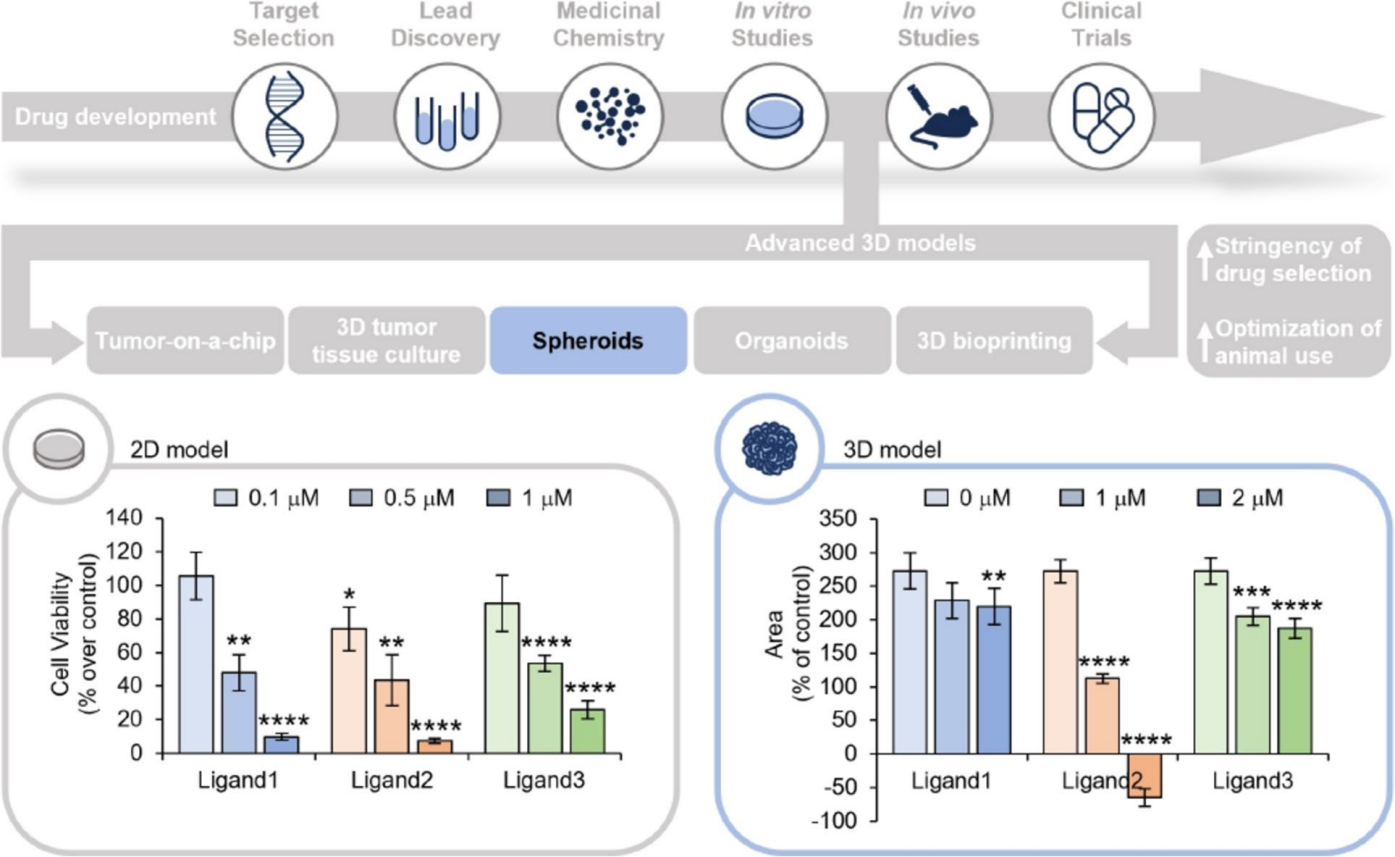
Upper panel. Schematic representation of drug development process. Bottom panels. Left panel: 2D cell viability assays performed after 48 h of treatment with the indicated compounds, at the final concentrations of 0.1, 0.5 and 1 μM. The results are expressed as the percentage of cell viability over the untreated cells. The histogram represents the mean values ± S.D. of three independent experiments. Right panel: Time course analysis of 3D tumor spheroids growth. Two days after the plating, the spheres were treated with the indicated compound at the final concentrations of 1 and 2 μM and monitored by Incucyte® S3 Live-Cell Analysis System (Essen BioScience, Ann Arbor, MI), 4X magnification. The results are expressed as the percentage of the spheroids area upon treatment relative to their own area before the compound administration. The graphs represent the mean values ± S.E.M. of at least 4 spheres. For each graph: *p < 0.05, **p < 0.01, ***p < 0.001, ****p < 0.0001

Drug development is a lengthy and complex process with a very low rate of success [32]. Notably, a key strategy to improve the entire process is represented by the implementation of the steps of biological validation. In this view, the development of 3D systems is assuming a particular relevance (Fig. 10, upper panel).

Here, we took advantages of spheroids, a typical advanced 3D in vitro model, for the screening of a library of potential anticancer molecules belonging to the family G-quadruplex (G4) ligands [33]. The comparison between the results obtained from canonical 2D cell lines and 3D models demonstrated that the use of spheroids, representing a model very close to tumor biology, increases the stringency of drug selection. Indeed, while we were able to identify three equally effective G4-ligands with cell cultures experiments, the results from 3D models analyses demonstrated that only one of these molecules was the most effective compound (Fig. 10, bottom panel). Moreover, as for patient tumor specimens, by processing the spheroids for immunohistochemical analyses (IHC), we were able to gain deeper insights in the molecular mechanisms underlying the tumor response to the new drug, directly in the context of tumor architectural complexity.

Our results defined 3D models as an essential tool that, linking the canonical 2D cell systems to preclinical animal models, would optimize the drug development process, limiting the number of candidate molecules for preclinical validation.

## hMENA-mediated autophagy in the dialogue among CAFs and cancer cells in NSCLC: organotypic tissue slices as a preclinical tool

*Annalisa Tocci* (IRCCS, Regina Elena National Cancer Institute, Rome, IT).

**Fig. 11:**
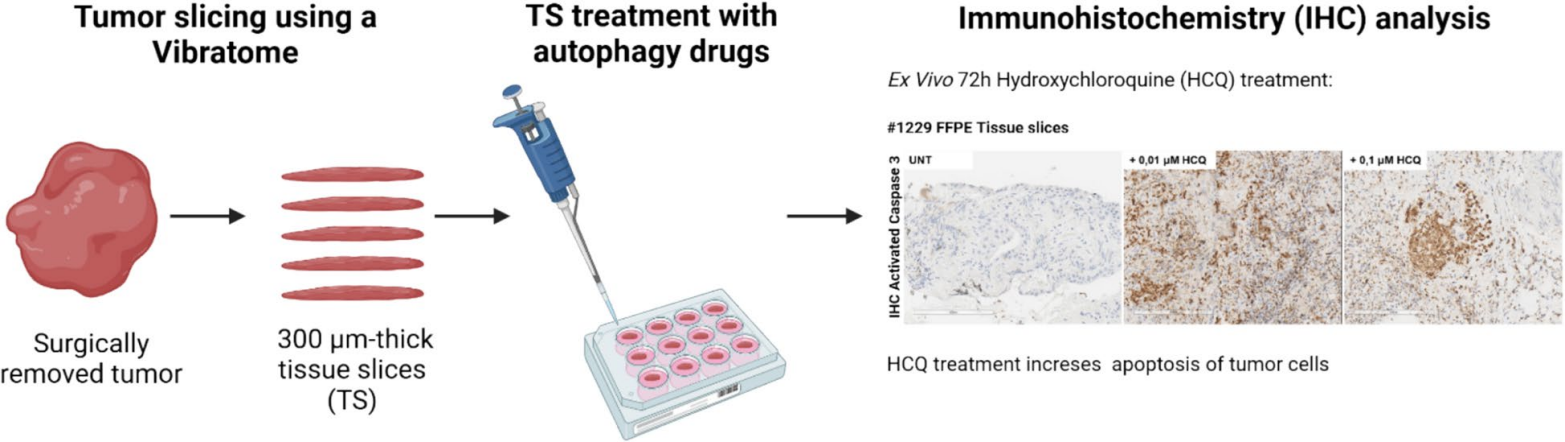
*Organotypic tissue slices as preclinical model to test autophagy drugs.* Illustration of the experimental workflow used in the study. Immunohistochemistry of activated Caspase 3 was used as a marker of apoptotic cell death. *Figure created with biorender.com*

The actin cytoskeleton regulatory protein hMENA and its tissue specific splicing-derived isoforms, differently affect non-small-cell lung cancer (NSCLC) prognosis and immunotherapy resistance [34–36]. hMENA and its isoform hMENAΔv6 are expressed in both mesenchymal tumor cells and CAFs [37]. Recently, a novel role for hMENA in autophagosome formation and trafficking within tumor cells has been identified [38]. Macroautophagy/autophagy, an evolutionarily conserved self-degradative and recycling process is essential for maintaining cellular function and homeostasis [39] also plays a role in unconventional protein secretion [40]. We investigated the role of hMENA isoforms in the communication between CAFs and cancer cells mediated by secretory autophagy. Using CAFs isolated from NSCLC tissues, we found that hMENA/hMENAΔv6 depletion impairs autophagy-mediated unconventional protein secretion. Conditioned medium from hMENA/hMENAΔv6 expressing CAFs affects NSCLC cell proliferation and glycolytic metabolism in an autophagy-dependent manner. We employed organotypic tissue slices, as preclinical model, to assess changes in cell viability after treatment with autophagy inhibitors (Fig. 11). In TCGA lung cancer patients a “secretory-autophagy gene signature” was enriched in hMENAΔv6^high^ patients. Our signature was also able to stratify responder and non-responder NSCLC patients treated with immune checkpoint inhibitors. These findings suggest a novel role for hMENA/hMENAΔv6 in autophagy-driven communication between CAFs and cancer cells, highlighting potential avenues for combinatorial therapies to broaden immunotherapy response.

## Identifying drug sensitivity of multifocal primary prostate cancer

*Sofia Karkampouna – Marianna Kruithof-de Julio* (University of Bern, Bern, CH)

The intra-patient heterogeneity observed in multifocal primary prostate cancer (PCa) has limited the effectiveness of existing treatment options (REF). Patient-derived organoids (PDOs) have emerged as a pivotal model for functional testing due to their capability to retain the histopathological and molecular characteristics of parental tissues, allowing timely acquisition of drug response outcomes. In our study, by employing twin biopsies from multiple lesions with matched PDO models in vitro, we investigated the molecular heterogeneity of PCa, and how it is linked to in vitro PDO pharmacological heterogeneity. Our functional testing approach leverages PDOs, as short-term primary cultures of self-assembling tumoroids in non-adherent and non extracellular matrix-containing conditions, of high tumor content and enrichment in luminal tumor cells [41]. PDOs screened with standard-of-care treatment and FDA-approved compounds for other malignancies, indicated receptor tyrosine kinase inhibitors are suitable for repurposing for PCa and therefore as possible alternatives to androgen deprivation therapy. By integrating gene expression data from parental tissue with drug response results from PDOs, we have established a transcriptomics-based drug prediction model using machine learning to identify responders and non-responders.

## Enhanced PARP inhibitor efficacy in 3D high-grade serous ovarian cancer models

*Piera Tocci* (IRCCS, Regina Elena National Cancer Institute, Rome, IT).

**Fig. 12:**
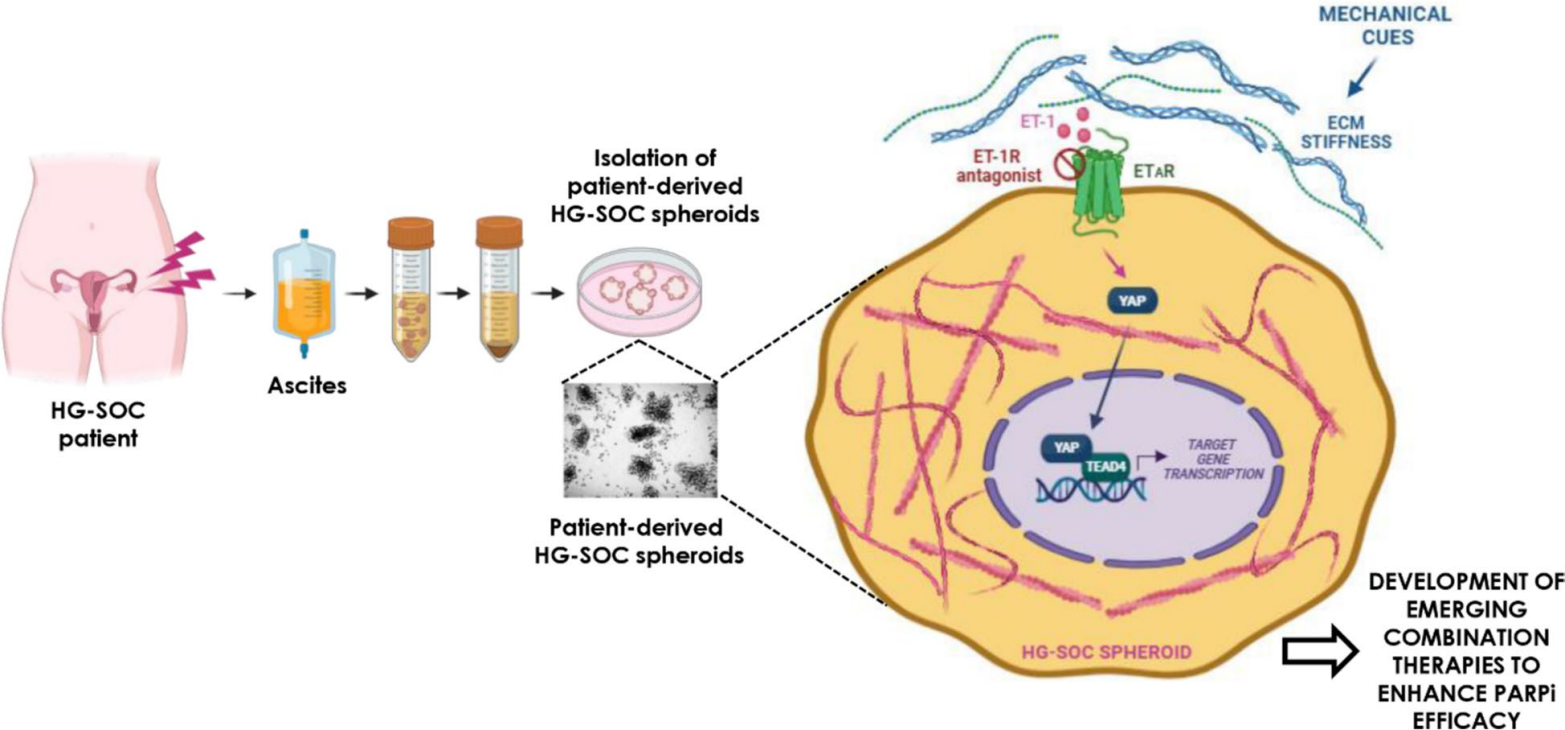
Graphic model depicting how in 3D high-grade serous ovarian cancer (HG-SOC) patient-derived spheroid models the ET-1/ET-1R/YAP circuit, intersecting mechanical forces generated by a stiff extracellular matrix (ECM), potentiates the HG-SOC invasive behaviour and sustains PARP inhibitors (PARPi) tolerance. *Figure created with biorender.com.* Abbreviations: EC_PDO: Patient-derived endometrial cancer organoids, 3D: Three-dimensional. PBMC: Peripheral blood mononuclear cells. TAMs: Tumor-associated macrophages. PDOs: Patient-derived organoids. HNSCC: Head and neck squamous cell carcinoma. ECM: Extracellular matrix

In the high-grade serous ovarian cancer (HG-SOC) ecosystem, high heterogeneous cancer cells, non-cancer cells and extracellular matrix (ECM) interact and evolve in complex and dynamic ways. Development of drugs such as poly ADP-ribose polymerase (PARP) inhibitors (PARPi) and targeted therapies are ineffective due to the onset of resistance. Among the molecular cues affecting PARPi response in HG-SOC there are those instigated by the endothelin-1 (ET-1) receptors (ET-1R) and intercepted by YAP [42]. However, how the mechanical forces contribute to PARPi failure is still unappreciated. In this framework, we leveraged HG-SOC patient-derived spheroids, able to resemble the HG-SOC features, including the 3D architecture and the mechanical pattern [43], proving that the ET-1/ET-1R system integrated the ECM-generated forces turning-on the YAP/TEAD transcriptional repertoire, strengthening HG-SOC invasiveness and impairing PARPi response. In HG-SOC PDX ET-1R blockade, potentiating PARPi success, halted the HG-SOC metastatic burden. Our study provides a 3D preclinical setting to explore and refine new HG-SOC treatment avenues (Fig. 12).

## Integrating spatial transcriptomics and organotypic tissue slices to unveil the Tumor Immune Microenvironment in Non-Small Cell Lung Cancer

Nicla Porciello (IRCCS, Regina Elena National Cancer Institute, Rome, IT).

**Fig. 13:**
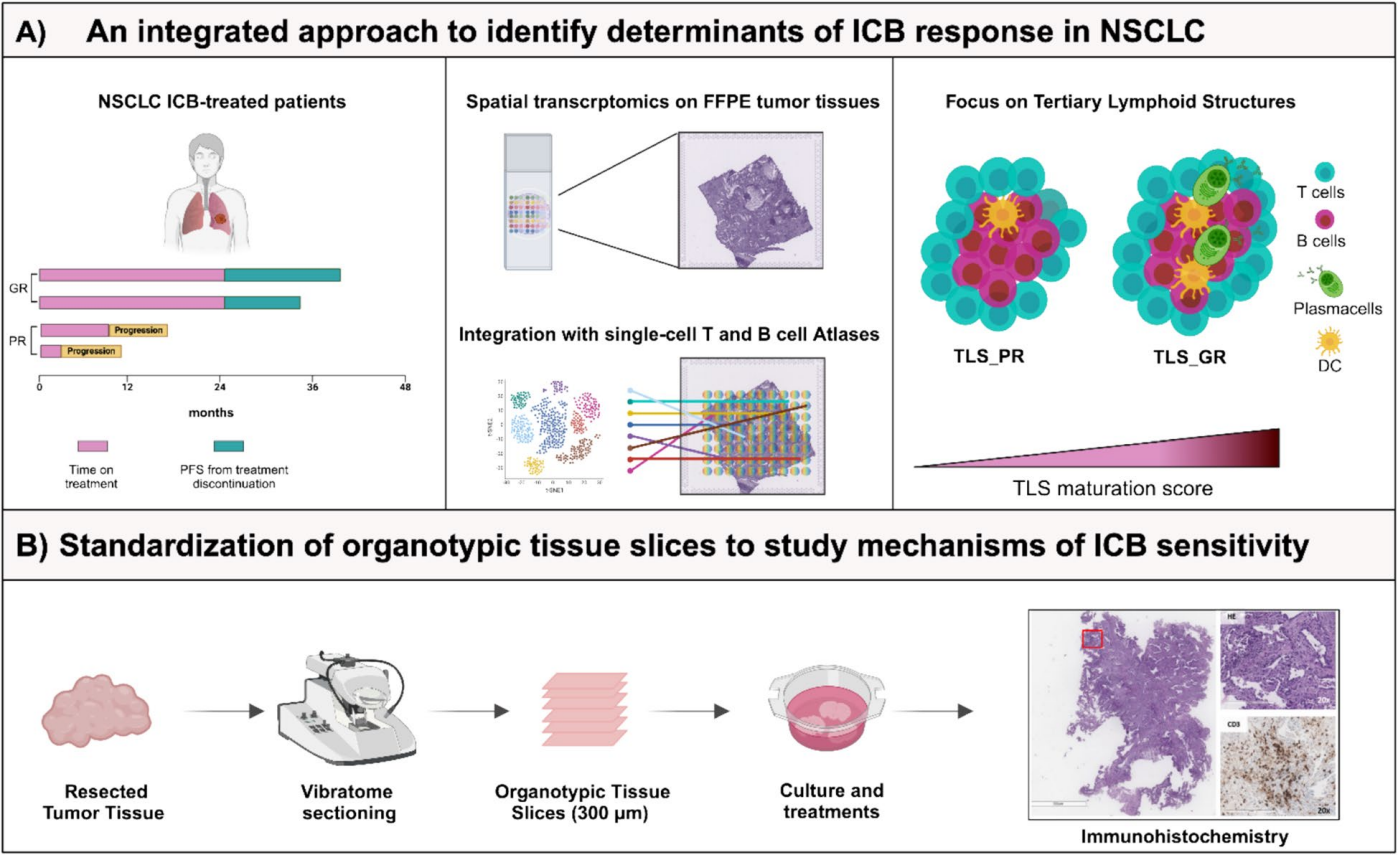
**A** Workflow of the integrated approach employed to identify determinants of ICB response. Archival tumor tissues, collected at the time of surgery, of a cohort of naïve of treatment NSCLC patients who recurred and were treated with ICB in accordance with guidelines were scored as poor (PR) and good (GR) ICB responders. Spatial transcriptomic by 10XVisium highlighted distinct composition and functionality of TLS of PR versus GR. **B** Standardization of organotypic tissue slices (TS) obtained from resected naïve of treatment NSCLC patients. TS were sliced, cultured, treated with ICB *ex-vivo* and then subjected to histological analysis. *Figure created with biorender.com*

The characterization and localization of the components of the Tumor Immune MicroEnvironment (TIME) are critical for determining clinical outcome in cancer patients. Tertiary Lymphoid Structures, organized immune hubs fostering T and B cell interactions to generate immune memory and high-affinity effector responses, have emerged as predictive biomarkers of response to Immune Checkpoint Blockade (ICB) [44]. Our laboratory identified a signature, comprising the actin regulatory protein hMENA, fibronectin, and lymphotoxin β receptor, as unexplored biomarker of TLS-enriched TIME, with prognostic and predictive value for ICB responsiveness in Non-Small Cell Lung Cancer (NSCLC) [34]. Here, by leveraging spatial transcriptomics on pre ICB-treated NSCLC tissues, integrated with single-cell atlases of T and B cells, we profiled the TLS transcriptome and highlighted increased TLS maturation score in Good versus poor ICB responder NSCLC patients, with increased abundance of activated-, mature-, switched-memory- and germinal center- B cells (Fig. 13A). To overcome the unavailability of post treatment-tumor tissues, we standardized organotypic tissue slices, from resected NSCLC specimens (Fig. 13B), preclinical models preserving both cellular and acellular TIME components in their original configuration [45]. We envisage that this integrated approach may pave the way for enhancing our understanding of immune evasion mechanisms and expanding the efficacy of ICB therapies.

## An organotypic 3D model to study the human liver fibrosis induction

*Klizia Maccaroni* (IRCCS, Regina Elena National Cancer Institute, Rome, IT).

**Fig. 14:**
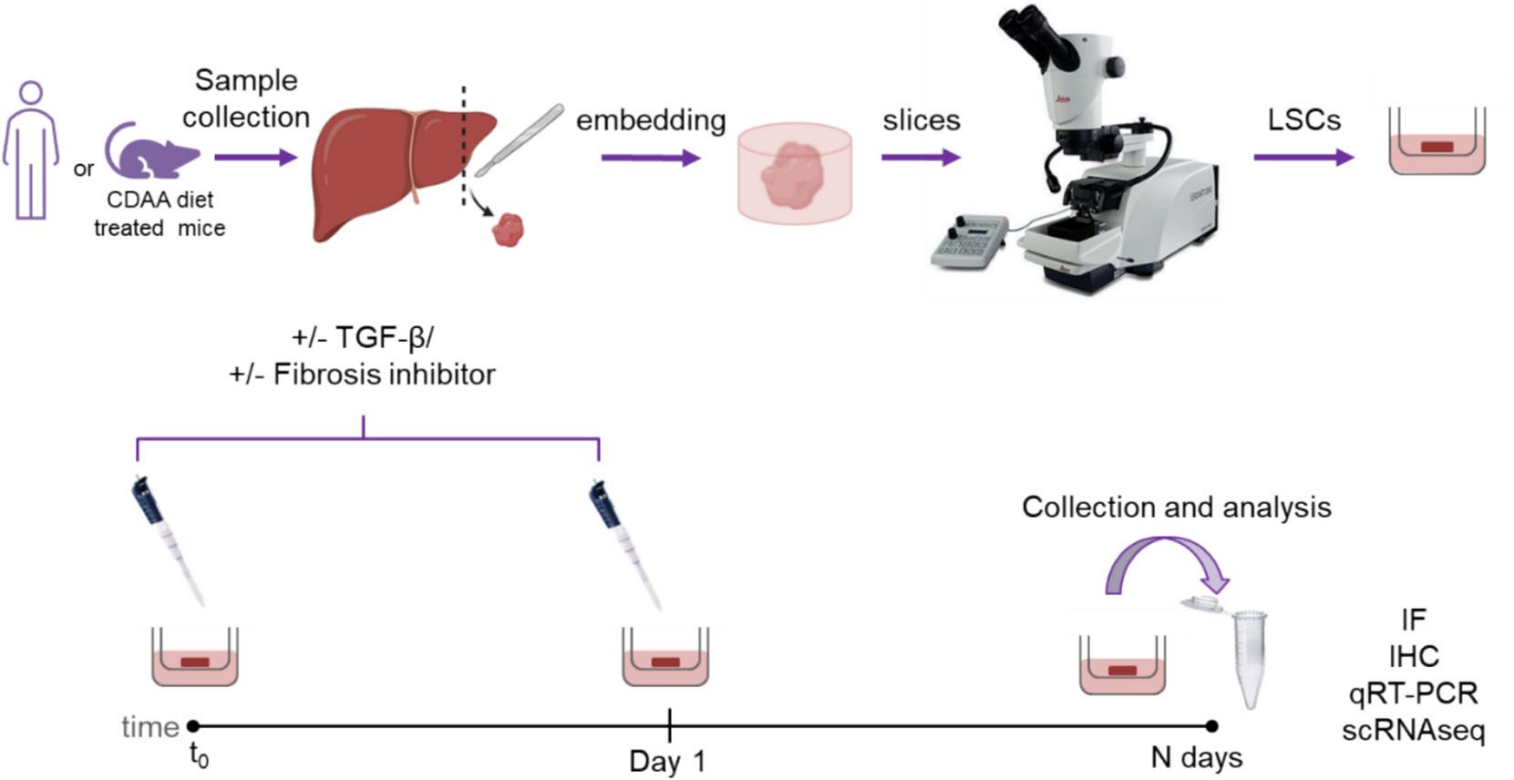
Specimens from healthy human livers or diet-treated mice are collected and cut to obtain 250 µm thick slices (Liver Slice Cultures, LSCs) before culturing them. TGF-β is added to human LSCs while pharmacological compounds are added to mouse LSCs. Slices are collected at different time points and the expression of pro-fibrotic markers analyzed

Liver fibrosis is the first step toward cirrhosis and a risk factor for hepatocellular carcinoma. It is caused by hepatic stellate cell differentiation into myofibroblasts and deposition of extracellular matrix. To identify markers for novel therapeutic strategies for fibrosis prevention and treatment, we established an organotypic model from healthy human livers based on previous protocols [46]. The increase of fibrotic markers after TGF-β treatment [47] suggests that our ex vivo model is suitable for studying human liver fibrosis. In addition, we generated a mouse organotypic model from murine hepatic tissue to study anti-fibrotic compounds. Liver slices from mice treated with a choline-deficient diet (CDAA) to induce non-alcoholic steatohepatitis were treated with GFT505 and BT173 [48]. Both drugs showed positive effects in downregulating the pro-fibrotic markers, confirming that the mouse organotypic CDAA model can be used for pharmacological screening of potential anti-fibrotic compounds (Fig. 14).

## Unraveling the complexity of metastatic breast cancer: insights from organoid cultures and multi-omics profiling

*Daniela Rutigliano* (IRCCS, Regina Elena National Cancer Institute, Rome, IT).

Breast cancer (BC) exhibits considerable heterogeneity, especially in advanced or metastatic cases (mBC), which complicates treatment options [49, 50]. Organoid cultures effectively model this complexity and aid the evaluation of mBC therapies [51, 52]. RNA sequencing (RNAseq) and whole-exome sequencing (WES) of BC metastases revealed unique gene signatures in BC metastases.

**Fig. 15:**
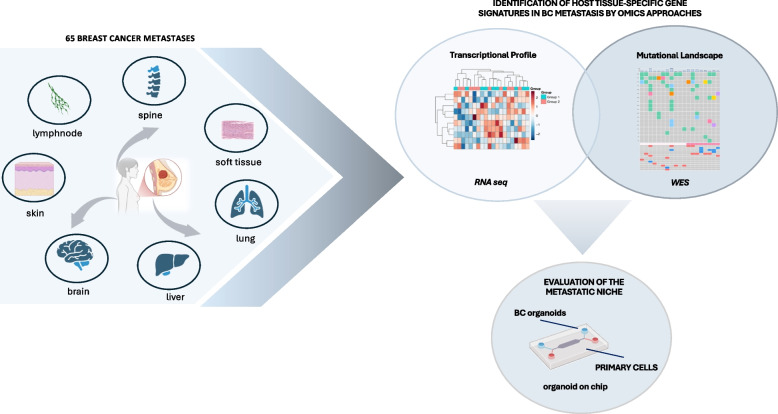
Breast Cancer Metastases Workflow

To study the heterogeneity of BC in vitro we generated organoids from both primary and metastatic BC tissues, confirming that these models accurately replicate the phenotypic and molecular characteristics of the original tumors. Additionally, to mimic the metastatic environment in vitro, we used organoid-on-chip technology to co-culture BC organoids with primary cells derived from the host tissue. This study sheds light on potential therapeutic strategies for metastatic BC, emphasizing the importance of tissue-specific approaches in treatment planning (Fig. 15).

*Xiling Shen* (Duke University, Durham, USA)

In an era increasingly defined by next-generation sequencing (NGS) and the burgeoning field of precision medicine, the question arose, "*Will organoids emerge as the quintessential technology for functional profiling and precision medicine?*" This paradigm shift underscores a crucial lesson for the advancement of tissue model technologies. Despite the technical prowess of many advanced tissue models, their application has remained niche due to inherent complexities. For a technology to transcend these limitations and achieve platform status, it must embody scalability. To elucidate the pathway to innovation in our field, we draw inspiration from the transformative principles observed in next-generation sequencing (NGS). The evolution of NGS embodies the power of miniaturization, where each sequencing reaction is scaled down to enable simultaneous capture of millions of reactions by high-speed imaging technology. This revolution has led to significant gains in speed, throughput, and accuracy, while simultaneously reducing costs through the "power of numbers" approach.

**Fig. 16:**
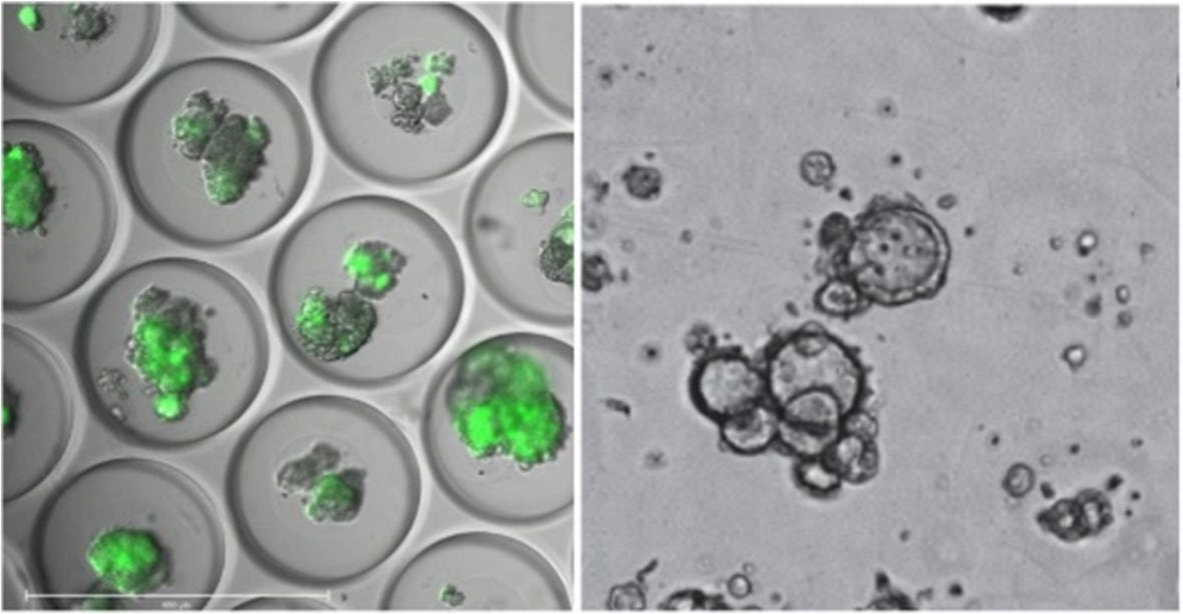
Left, GFP-labeled MOs in micro-wells. Right, Patient-derived MOs in a well-plate, ready for drug testing

The cornerstone of the presented platform comprises of micro-organoids (MOs) via emulsion microfluidics [1, 53, 54] (Fig. 16). Data were further presented to demonstrate their key differentiators compared to existing technologies, including: (1) *Rapid Analysis from Minimal Tissue Samples*: The technology facilitates a swift turnaround for drug assays, delivering reliable results in under two weeks from tissue samples as small as those obtained from an 18-gauge biopsy; (2) *Enhanced Throughput and Cost Efficiency*: The technology supports high-throughput screening, leveraging standard liquid handling and imaging equipment to optimize both throughput and cost-effectiveness; (3) *Preservation of Tissue Microenvironment*: the technology is adept at maintaining the native tissue microenvironment (TME), preserving crucial components such as stromal and immune cells across diverse tissue types, thereby enhancing the biological relevance of the assays.

Building on these strengths, case studies were presented showcasing how MO has been driving innovation in therapeutic development through 'patient MO avatar trials,' accelerating the clinical development of immunotherapy drugs via 'smart clinical trials,' and advancing functional precision medicine to optimize treatment decisions for patients.

## Data Availability

No datasets were generated or analysed during the current study.

## Abbreviations

PDAC: Pancreatic ductal adenocarcinoma
TME: Tumor microenvironment
PDCs: Patient-derived cells
PFP: Personalized Functional Profiling
mCRC: Metastatic colorectal cancer
rGBM: Recurrent glioblastomas
AUC: Area under curve
DRC: Dose response curve
RT: Rare tumors
TIICs: Tumor infiltrating immune cells
RC: Radical cystectomy
TURBT: Transurethral resection of bladder tumor
iCCA: Intrahepatic cholangiocarcinoma
FFPs: Fusion proteins
OS: Osteosarcoma
G4: G-quadruplex
IHC: Immunohistochemical analyses
NSCLC: Non-small-cell lung cancer NSCLC
PC: Prostate cancer
PARP: Poly ADP-ribose polymerase
ET-1: Endothelin-1
TIME: Tumor Immune MicroEnvironment
ICB: Immune Checkpoint Blockade
CDAA: Choline-deficient diet
BC: Breast cancer
RNAseq: RNA sequencing
WES: Whole-exome sequencing
NGS: Next-generation sequencing

## References

[1] Bose S, Barroso M, Chheda MG, Clevers H, Elez E, Kaochar S, Kopetz SE, Li XN, Meric-Bernstam F, Meyer CA, et al. A path to translation: How 3D patient tumor avatars enable next generation precision oncology. Cancer Cell. 2022;40:1448–53. 36270276 10.1016/j.ccell.2022.09.017 PMC10576652

[2] de Visser KE, Joyce JA. The evolving tumor microenvironment: From cancer initiation to metastatic outgrowth. Cancer Cell. 2023;41:374–403. 36917948 10.1016/j.ccell.2023.02.016

[3] Neufeld L, Yeini E, Pozzi S, Satchi-Fainaro R. 3D bioprinted cancer models: from basic biology to drug development. Nat Rev Cancer. 2022;22:679–92. 36280768 10.1038/s41568-022-00514-w

[4] Neufeld L, Yeini E, Reisman N, Shtilerman Y, Ben-Shushan D, Pozzi S, Madi A, Tiram G, Eldar-Boock A, Ferber S, et al: Microengineered perfusable 3D-bioprinted glioblastoma model for in vivo mimicry of tumor microenvironment. Sci Adv. 2021;7(34):eabi9119. 10.1126/sciadv.abi9119 PMC8373143 34407932

[5] Yeini E, Ofek P, Pozzi S, Albeck N, Ben-Shushan D, Tiram G, Golan S, Kleiner R, Sheinin R, Israeli Dangoor S, et al. P-selectin axis plays a key role in microglia immunophenotype and glioblastoma progression. Nat Commun. 1912;2021:12. 10.1038/s41467-021-22186-0 PMC7997963 33771989

[6] Yan Y, Shi P, Song W, Bi S. Chemiluminescence and Bioluminescence Imaging for Biosensing and Therapy: In Vitro and In Vivo Perspectives. Theranostics. 2019;9:4047–65. 31281531 10.7150/thno.33228 PMC6592176

[7] Hsu KS, Adileh M, Martin ML, Makarov V, Chen J, Wu C, Bodo S, Klingler S, Sauve CG, Szeglin BC, et al. Colorectal Cancer Develops Inherent Radiosensitivity That Can Be Predicted Using Patient-Derived Organoids. Cancer Res. 2022;82:2298–312. 35472075 10.1158/0008-5472.CAN-21-4128 PMC9390071

[8] Cancer Genome Atlas N. Comprehensive genomic characterization of head and neck squamous cell carcinomas. Nature. 2015;517:576–82. 25631445 10.1038/nature14129 PMC4311405

[9] Pierik AS, Poell JB, Brink A, Stigter-van Walsum M, de Roest RH, Poli T, Yaromin A, Lambin P, Leemans CR, Brakenhoff RH. Intratumor genetic heterogeneity and head and neck cancer relapse. Radiother Oncol. 2024;191:110087. 38185257 10.1016/j.radonc.2024.110087

[10] Budach V, Tinhofer I. Novel prognostic clinical factors and biomarkers for outcome prediction in head and neck cancer: a systematic review. Lancet Oncol. 2019;20:e313–26. 31162105 10.1016/S1470-2045(19)30177-9

[11] Fisch AS, Pestana A, Sachse V, Doll C, Hofmann E, Heiland M, Obermueller T, Heidemann J, Dommerich S, Schoppe D, et al. Feasibility analysis of using patient-derived tumour organoids for treatment decision guidance in locally advanced head and neck squamous cell carcinoma. Eur J Cancer. 2024;213:115100. 39476443 10.1016/j.ejca.2024.115100

[12] Tedesco M, Giannese F, Lazarevic D, Giansanti V, Rosano D, Monzani S, Catalano I, Grassi E, Zanella ER, Botrugno OA, et al. Chromatin Velocity reveals epigenetic dynamics by single-cell profiling of heterochromatin and euchromatin. Nat Biotechnol. 2022;40:235–44. 34635836 10.1038/s41587-021-01031-1

[13] De Stefano P, Bianchi E, Dubini G. The impact of microfluidics in high-throughput drug-screening applications. Biomicrofluidics. 2022;16:031501. 35646223 10.1063/5.0087294 PMC9142169

[14] Northcott JM, Dean IS, Mouw JK, Weaver VM. Feeling Stress: The Mechanics of Cancer Progression and Aggression. Front Cell Dev Biol. 2018;6:17. 29541636 10.3389/fcell.2018.00017 PMC5835517

[15] LeSavage BL, Suhar RA, Broguiere N, Lutolf MP, Heilshorn SC. Next-generation cancer organoids. Nat Mater. 2022;21:143–59. 34385685 10.1038/s41563-021-01057-5 PMC12276900

[16] LeSavage BL, Zhang D, Huerta-Lopez C, Gilchrist AE, Krajina BA, Karlsson K, Smith AR, Karagyozova K, Klett KC, Huang MS, et al. Engineered matrices reveal stiffness-mediated chemoresistance in patient-derived pancreatic cancer organoids. Nat Mater. 2024;23:1138–49. 38965405 10.1038/s41563-024-01908-x PMC13098013

[17] Rulten SL, Grose RP, Gatz SA, Jones JL, Cameron AJM: The Future of Precision Oncology. Int J Mol Sci. 2023;24(16):12613. 10.3390/ijms241612613 PMC10454858 37628794

[18] Malone ER, Oliva M, Sabatini PJB, Stockley TL, Siu LL. Molecular profiling for precision cancer therapies. Genome Med. 2020;12:8. 31937368 10.1186/s13073-019-0703-1 PMC6961404

[19] De La Acanda Rocha AM, Berlow NE, Fader M, Coats ER, Saghira C, Espinal PS, Galano J, Khatib Z, Abdella H, Maher OM, et al. Feasibility of functional precision medicine for guiding treatment of relapsed or refractory pediatric cancers. Nat Med. 2024;30:990–1000. 38605166 10.1038/s41591-024-02848-4 PMC11031400

[20] Lee JK, Liu Z, Sa JK, Shin S, Wang J, Bordyuh M, Cho HJ, Elliott O, Chu T, Choi SW, et al. Pharmacogenomic landscape of patient-derived tumor cells informs precision oncology therapy. Nat Genet. 2018;50:1399–411. 30262818 10.1038/s41588-018-0209-6 PMC8514738

[21] Gatta G, van der Zwan JM, Casali PG, Siesling S, Dei Tos AP, Kunkler I, Otter R, Licitra L, Mallone S, Tavilla A, et al. Rare cancers are not so rare: the rare cancer burden in Europe. Eur J Cancer. 2011;47:2493–511. 22033323 10.1016/j.ejca.2011.08.008

[22] Habanjar O, Diab-Assaf M, Caldefie-Chezet F, Delort L: 3D Cell Culture Systems: Tumor Application, Advantages, and Disadvantages. Int J Mol Sci. 2021;22(22):12200. 10.3390/ijms222212200 PMC8618305 34830082

[23] Fiorini E, Veghini L, Corbo V. Modeling Cell Communication in Cancer With Organoids: Making the Complex Simple. Front Cell Dev Biol. 2020;8:166. 32258040 10.3389/fcell.2020.00166 PMC7094029

[24] Dyrskjot L, Hansel DE, Efstathiou JA, Knowles MA, Galsky MD, Teoh J, Theodorescu D. Bladder cancer Nat Rev Dis Primers. 2023;9:58. 37884563 10.1038/s41572-023-00468-9 PMC11218610

[25] Meeks JJ, Al-Ahmadie H, Faltas BM, Taylor JA 3rd, Flaig TW, DeGraff DJ, Christensen E, Woolbright BL, McConkey DJ, Dyrskjot L. Genomic heterogeneity in bladder cancer: challenges and possible solutions to improve outcomes. Nat Rev Urol. 2020;17:259–70. 32235944 10.1038/s41585-020-0304-1 PMC7968350

[26] Medle B, Sjodahl G, Eriksson P, Liedberg F, Hoglund M, Bernardo C: Patient-Derived Bladder Cancer Organoid Models in Tumor Biology and Drug Testing: A Systematic Review. Cancers (Basel). 2022;14(9):2062. 10.3390/cancers14092062 PMC9104249 35565191

[27] Radic M, Egger M, Kruithof-de Julio M, Seiler R: Patient-derived Organoids in Bladder Cancer: Opportunities and Challenges. Eur Urol Focus. 2024:S2405-4569(24)00165-2. 10.1016/j.euf.2024.08.008 39232905

[28] Lowery MA, Ptashkin R, Jordan E, Berger MF, Zehir A, Capanu M, Kemeny NE, O’Reilly EM, El-Dika I, Jarnagin WR, et al. Comprehensive Molecular Profiling of Intrahepatic and Extrahepatic Cholangiocarcinomas: Potential Targets for Intervention. Clin Cancer Res. 2018;24:4154–61. 29848569 10.1158/1078-0432.CCR-18-0078 PMC6642361

[29] Cristinziano G, Porru M, Lamberti D, Buglioni S, Rollo F, Amoreo CA, Manni I, Giannarelli D, Cristofoletti C, Russo G, et al. FGFR2 fusion proteins drive oncogenic transformation of mouse liver organoids towards cholangiocarcinoma. J Hepatol. 2021;75:351–62. 33741397 10.1016/j.jhep.2021.02.032

[30] Kansara M, Teng MW, Smyth MJ, Thomas DM. Translational biology of osteosarcoma. Nat Rev Cancer. 2014;14:722–35. 25319867 10.1038/nrc3838

[31] Smith HL, Beers SA, Gray JC, Kanczler JM: The Role of Pre-Clinical 3-Dimensional Models of Osteosarcoma. Int J Mol Sci. 2020;21(15):5499. 10.3390/ijms21155499 PMC7432883 32752092

[32] Sun D, Gao W, Hu H, Zhou S. Why 90% of clinical drug development fails and how to improve it? Acta Pharm Sin B. 2022;12:3049–62. 35865092 10.1016/j.apsb.2022.02.002 PMC9293739

[33] Iachettini S, Biroccio A, Zizza P: Therapeutic Use of G4-Ligands in Cancer: State-of-the-Art and Future Perspectives. Pharmaceuticals (Basel). 2024;17(6):771. 10.3390/ph17060771 PMC11206494 38931438

[34] Di Modugno F, Di Carlo A, Spada S, Palermo B, D’Ambrosio L, D’Andrea D, Morello G, Belmonte B, Sperduti I, Balzano V, et al. Tumoral and stromal hMENA isoforms impact tertiary lymphoid structure localization in lung cancer and predict immune checkpoint blockade response in patients with cancer. EBioMedicine. 2024;101:105003. 38340557 10.1016/j.ebiom.2024.105003 PMC10869748

[35] Trono P, Tocci A, Palermo B, Di Carlo A, D'Ambrosio L, D'Andrea D, Di Modugno F, De Nicola F, Goeman F, Corleone G, et al: hMENA isoforms regulate cancer intrinsic type I IFN signaling and extrinsic mechanisms of resistance to immune checkpoint blockade in NSCLC. Cancer. 2023;11(8):e006913. 10.1136/jitc-2023-006913. 10.1136/jitc-2023-006913 PMC10450042 37612043

[36] Di Modugno F, Spada S, Palermo B, Visca P, Iapicca P, Di Carlo A, Antoniani B, Sperduti I, Di Benedetto A, Terrenato I, et al. hMENA isoforms impact NSCLC patient outcome through fibronectin/beta1 integrin axis. Oncogene. 2018;37:5605–17. 29907768 10.1038/s41388-018-0364-3 PMC6193944

[37] Melchionna R, Spada S, Di Modugno F, D’Andrea D, Di Carlo A, Panetta M, Mileo AM, Sperduti I, Antoniani B, Gallo E, et al. The actin modulator hMENA regulates GAS6-AXL axis and pro-tumor cancer/stromal cell cooperation. EMBO Rep. 2020;21:e50078. 32909687 10.15252/embr.202050078 PMC7645265

[38] Li Y, Zhang Y, Wang M, Su J, Dong X, Yang Y, Wang H, Li Q. The mammalian actin elongation factor ENAH/MENA contributes to autophagosome formation via its actin regulatory function. Autophagy. 2024;20:1798–814. 38705725 10.1080/15548627.2024.2347105 PMC11262208

[39] Piletic K, Alsaleh G, Simon AK. Autophagy orchestrates the crosstalk between cells and organs. EMBO Rep. 2023;24:e57289. 37465980 10.15252/embr.202357289 PMC10481659

[40] New J, Thomas SM. Autophagy-dependent secretion: mechanism, factors secreted, and disease implications. Autophagy. 2019;15:1682–93. 30894055 10.1080/15548627.2019.1596479 PMC6735501

[41] Karkampouna S, La Manna F, Benjak A, Kiener M, De Menna M, Zoni E, Grosjean J, Klima I, Garofoli A, Bolis M, et al. Patient-derived xenografts and organoids model therapy response in prostate cancer. Nat Commun. 2021;12:1117. 33602919 10.1038/s41467-021-21300-6 PMC7892572

[42] Tocci P, Roman C, Sestito R, Di Castro V, Sacconi A, Molineris I, Paolini F, Carosi M, Tonon G, Blandino G, Bagnato A. Targeting tumor-stroma communication by blocking endothelin-1 receptors sensitizes high-grade serous ovarian cancer to PARP inhibition. Cell Death Dis. 2023;14:5. 36604418 10.1038/s41419-022-05538-6 PMC9816119

[43] Lopez E, Kamboj S, Chen C, Wang Z, Kellouche S, Leroy-Dudal J, Carreiras F, Lambert A, Aime C: In Vitro Models of Ovarian Cancer: Bridging the Gap between Pathophysiology and Mechanistic Models. Biomolecules 2023;13. 10.3390/biom13010103 PMC9855568 36671488

[44] Fridman WH, Meylan M, Pupier G, Calvez A, Hernandez I, Sautes-Fridman C. Tertiary lymphoid structures and B cells: An intratumoral immunity cycle. Immunity. 2023;56:2254–69. 37699391 10.1016/j.immuni.2023.08.009

[45] Kenerson HL, Sullivan KM, Labadie KP, Pillarisetty VG, Yeung RS. Protocol for tissue slice cultures from human solid tumors to study therapeutic response. STAR Protoc. 2021;2:100574. 34142099 10.1016/j.xpro.2021.100574 PMC8184656

[46] Jiang X, Seo YD, Sullivan KM, Pillarisetty VG. Establishment of Slice Cultures as a Tool to Study the Cancer Immune Microenvironment. Methods Mol Biol. 2019;1884:283–95. 30465211 10.1007/978-1-4939-8885-3_20

[47] Woodcock HV, Eley JD, Guillotin D, Plate M, Nanthakumar CB, Martufi M, Peace S, Joberty G, Poeckel D, Good RB, et al. The mTORC1/4E-BP1 axis represents a critical signaling node during fibrogenesis. Nat Commun. 2019;10:6. 30602778 10.1038/s41467-018-07858-8 PMC6315032

[48] Scaglione A, Monteonofrio L, Parisi G, Cecchetti C, Siepi F, Rinaldo C, Giorgi A, Verzili D, Zamparelli C, Savino C, et al. Effects of Y361-auto-phosphorylation on structural plasticity of the HIPK2 kinase domain. Protein Sci. 2018;27:725–37. 29277937 10.1002/pro.3367 PMC5818748

[49] Turner KM, Yeo SK, Holm TM, Shaughnessy E, Guan JL. Heterogeneity within molecular subtypes of breast cancer. Am J Physiol Cell Physiol. 2021;321:C343–54. 34191627 10.1152/ajpcell.00109.2021 PMC8424677

[50] Luond F, Tiede S, Christofori G. Breast cancer as an example of tumour heterogeneity and tumour cell plasticity during malignant progression. Br J Cancer. 2021;125:164–75. 33824479 10.1038/s41416-021-01328-7 PMC8292450

[51] Donzelli S, Cioce M, Sacconi A, Zanconato F, Daralioti T, Goeman F, Orlandi G, Di Martino S, Fazio VM, Alessandrini G, et al. A PIK3CA-mutant breast cancer metastatic patient-derived organoid approach to evaluate alpelisib treatment for multiple secondary lesions. Mol Cancer. 2022;21:152. 35869553 10.1186/s12943-022-01617-6 PMC9306102

[52] Tzeng YT, Hsiao JH, Tseng LM, Hou MF, Li CJ. Breast cancer organoids derived from patients: A platform for tailored drug screening. Biochem Pharmacol. 2023;217:115803. 37709150 10.1016/j.bcp.2023.115803

[53] Ding S, Hsu C, Wang Z, Natesh NR, Millen R, Negrete M, Giroux N, Rivera GO, Dohlman A, Bose S, et al. Patient-derived micro-organospheres enable clinical precision oncology. Cell Stem Cell. 2022;29(905–917):e906. 10.1016/j.stem.2022.04.006 PMC9177814 35508177

[54] Wang Z, Boretto M, Millen R, Natesh N, Reckzeh ES, Hsu C, Negrete M, Yao H, Quayle W, Heaton BE, et al. Rapid tissue prototyping with micro-organospheres. Stem Cell Reports. 2022;17:1959–75. 35985334 10.1016/j.stemcr.2022.07.016 PMC9481922

